# Therapeutic gene editing of T cells corrects CTLA4 insufficiency

**DOI:** 10.1126/scitranslmed.abn5811

**Published:** 2022-10-26

**Authors:** TA Fox, BC Houghton, L Petersone, E Waters, NM Edner, A McKenna, O Preham, C Hinze, C Williams, AS Albuquerque, A Kennedy, AM Pesenacker, P Genovese, LSK Walker, SO Burns, DM Sansom, C Booth, EC Morris

**Affiliations:** 1 UCL Institute of Immunity and Transplantation, UCL, London, UK; 2 Department of Haematology, University College London NHS Foundation Trust, London, UK; 3 UCL Great Ormond Street Institute of Child Health, UCL, London, UK; 4 Dana-Farber/Boston Children’s Cancer and Blood Disorder Center, Boston, USA; 5 Department Immunology, Royal Free London Hospitals NHS Foundation Trust, London, UK; 6 Department of Paediatric Immunology, Great Ormond Street Hospital, London, UK; 7 UCLH NIHR Biomedical Research Centre, London, UK

## Abstract

Heterozygous mutations in *CTLA4* result in an inborn error of immunity (IEI) with an autoimmune and frequently severe clinical phenotype. Autologous T cell gene therapy may offer a cure without the immunological complications of allogeneic stem cell transplantation. We designed a homology directed repair (HDR) gene editing strategy that inserts the *CTLA4* cDNA into the first intron of the *CTLA4* genomic locus. This resulted in regulated expression of CTLA4 in CD4+ T cells and functional studies demonstrated CD80 and CD86 transendocytosis. Gene editing of T cells isolated from patients with CTLA4 insufficiency also restored CTLA4 expression and rescued transendocytosis of CD80 and CD86 *in vitro*. Finally, gene corrected T cells from *CTLA4* -/- mice engrafted and prevented lymphoproliferation in an *in vivo* murine model of CTLA4 insufficiency. These results demonstrate the feasibility of a novel therapeutic approach using T cell gene therapy for CTLA4 insufficiency.

## Introduction

**Figure 1:**
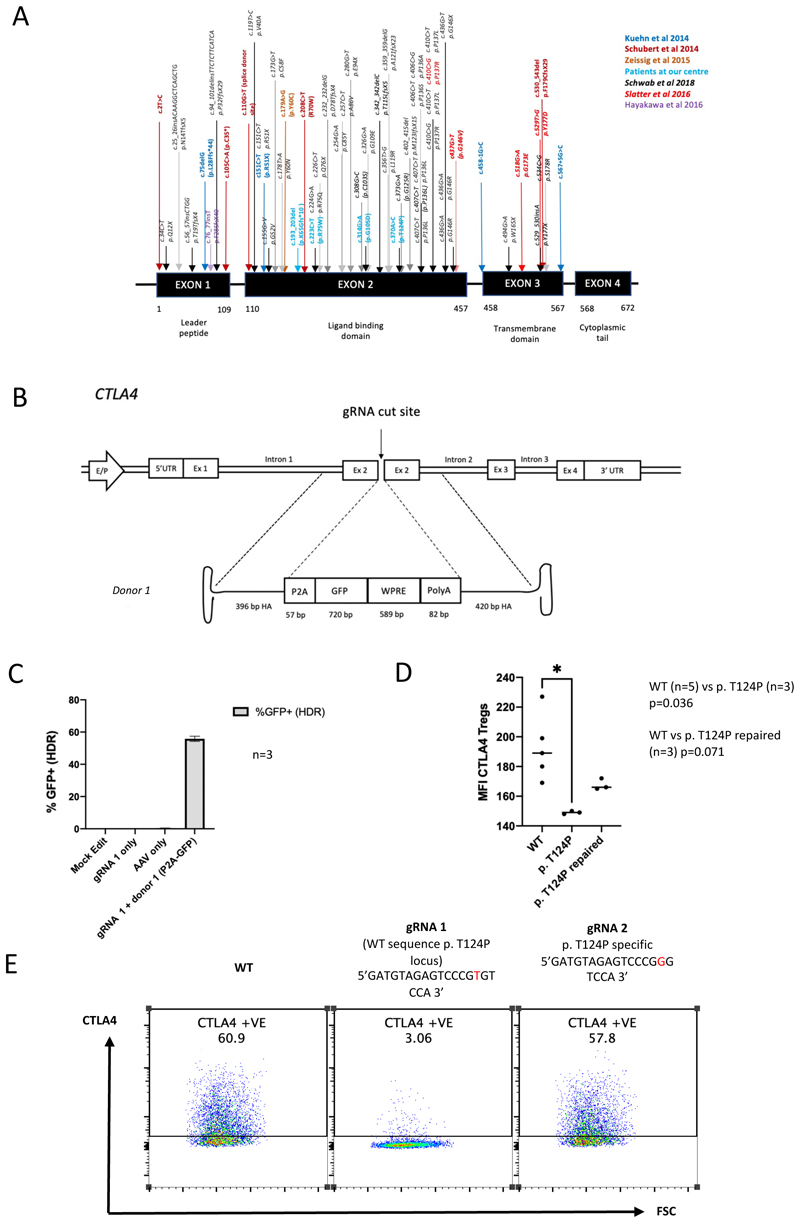
Targeting the *CTLA4* locus with CRISPR/Cas9 and repair of a point mutation (A) Schematic representation of the mutational landscape of CTLA4 insufficiency. Mutations are colour coded by citation (key, bottom right). (B) Schematic representation of HDR donor 1 (P2A-GFP-WPRE-PolyA). (C) Average HDR rate (n=3, percentage GFP+ in cells from separate healthy donors) (Mean 55.83 SD 1.626). (D) Median fluorescent intensity (MFI) of CTLA4 from 5 separate healthy controls and 3 separate samples from a single patient with p.T124P c.370A>C unedited or edited. A significant difference was seen in CTLA4 MFO between WT and p. T124P het mutant cells (p=0.036, Man Whitney test). After editing the difference in MFI was no longer significant. (E) Flow cytometry plot demonstrating surface CTLA4 expression in cells from a healthy individual in an unedited control (left), edited with a gRNA specific for the wild type CTLA4 sequence (gRNA 1) with resulting knock down of CTLA4 protein (centre) and a on edited with a gRNA specific for the p.T124P c.371A>C (gRNA 2) (right) demonstrating minimal activity on the wild-type sequence.

CTLA4 insufficiency is an inborn error of immunity (IEI) (primary immunodeficiency) with a severe clinical phenotype that results from heterozygous germline mutations in *CTLA4*.^1,2^ CTLA4 insufficiency is a new diagnosis, first described in 2014.^1–3^ Many patients had a previous diagnosis of common variable immune deficiency (CVID) and/or an autoimmune syndrome, however as availability of genetic diagnosis and awareness of the condition has improved the number of recognized cases has increased dramatically.^4,5^ CTLA4 insufficiency has a heterogeneous genetic landscape with no obvious hotspots, although most disease-causing mutations (>80%) are found in exons 2 and 3 (Figure 1A).^1–3,5–8^

CTLA4 (CD152) is a critical negative immune regulator, expressed constitutively on regulatory T cells (Tregs) and on conventional T cells (Tconv) upon activation. ^9,10^ CTLA4 competes with CD28, for the shared ligands CD80 and CD86, expressed on antigen presenting cells (APCs). CTLA4 binds its ligands and then removes them from APCs by the process of transendocytosis (TE) thereby depleting the same ligands required for CD28 co-stimulation, causing immunosupression.^3,10,11^ Patients with CTLA4 insufficiency can exhibit an increased percentage of CD4+ FOXP3+ Treg cells compared to healthy controls, ^2,3^ however within the Treg fraction, lower CTLA4 expression and a reduction in ligand uptake (CD80 and CD86) has been observed, consistent with compromised Treg function.^2,12^

Clinically, CTLA4 insufficiency is characterized by immune dysregulation due to reduced suppression by Tregs and consequent hyperactivation of effector T cells.^1,2^ It presents in the first five decades of life with hypogammaglobulinemia, recurrent infections, profound autoimmunity and lymphoproliferation (which can be malignant) resulting in progressive morbidity and premature mortality.^1,3,13^ Management is challenging and whilst the CTLA4 fusion protein mimetics (abatacept and belatacept) can result in clinical improvement, concomitant systemic immunosuppression is usually required to control autoimmunity.^3,13,14^ Allogeneic hematopoietic stem cell transplantation (alloHSCT) is therefore currently the only curative treatment however, it carries high risk of mortality as well as morbidity from graft failure, graft rejection and graft-versus-host disease (GVHD).^3,8,15^

Autologous gene therapy (GT) is a potential curative approach without the immunological complications of alloHSCT. Gammaretroviral and lentiviral hematopoietic stem cell (HSC) GT has been successfully used to treat other IEIs by introducing a transgene which integrates semi-randomly into the genome with expression driven by an artificial promoter.^16–22^ However, in disorders where tightly regulated gene expression is required, as is the case in gain-of-function disorders and haploinsufficiency such as CTLA4 insufficiency, a specific gene editing approach may be more appropriate facilitating physiological, dynamic, cell-specific protein expression.

Gene editing technologies such as the clustered regularly interspaced short palindromic repeats-associated protein 9 (CRISPR-Cas9) system, enable correction of genetic defects whilst preserving the endogenous gene control machinery.^23^ New genetic material can be inserted by the process of homology directed repair (HDR) at the site of a double-stranded DNA (dsDNA) break using non-integrating templates such as adeno-associated virus 6 (AAV6) vectors.^24^ Several published pre-clinical studies have demonstrated that IEIs can be corrected using gene editing, thus providing proof-of-principle of autologous GT approaches for these disorders.^25–30^ Gene edited cellular therapies have entered the clinic for the treatment of monogenic disorders with promising results published for sickle cell disease and beta-thalassaemia.^31^

Most gene therapy approaches for IEIs modify hematopoietic stem cells (HSCs) in order to achieve gene expression across all hematopoietic lineages.^19,20,22^ However, for conditions primarily affecting the lymphoid compartment such as CD40 ligand deficiency, X-linked lymphoproliferative disease and CTLA4 insufficiency, restoration of T cell function may offer a cure.^3,25,32,33^ T cell GT has several advantages over HSC-GT. Firstly, large numbers of T-lymphocytes can be obtained with non-mobilised apheresis. The lymphodepletion required prior to infusion of a T-cell product is significantly less toxic than the myeloablative regimens required for HSC engraftment. As T-cells are terminally differentiated, the risk of insertional mutagenesis is reduced.^32^ Although the pathophysiology of CTLA4 insufficiency is not strictly confined to the T cell compartment, CTLA4 functions in a cell-extrinsic manner thus we hypothesize that T cell correction could significantly abrogate the clinical phenotype.^11^

Here, we report a widely applicable gene editing approach that corrects the immunological defect in CTLA4 insufficient T cells. We demonstrate functional restoration in patient T cells *in vitro* and abrogation of the clinical phenotype in an *in vivo* murine model of the disease. Our data demonstrate the feasibility of a novel therapeutic approach using T cell gene therapy for CTLA4 insufficiency.

## Results

### Targeted genome modification of the human CTLA4 locus using CRISPR/Cas9 and AAV HDR template

To demonstrate the feasibility of performing gene editing at the *CTLA4* locus and to optimize our editing protocol in human T cells we designed a CRISPR/Cas9/AAV6 approach that inserted a green fluorescent protein (GFP) sequence into the open reading frame (ORF) of *CTLA4*. We used a 20-nucleotide guide RNA (gRNA) (gRNA 1; GAUGUAGAGUCCCGUGUCCA) that produced a dsDNA break in exon 2 of *CTLA4* (Supplementary Figure 1A). This break was targeted for repair using an AAV6 donor template incorporating a promoterless 2A self-cleaving peptide (P2A) – green-fluorescent protein (GFP) sequence followed by a synthetic woodchuck hepatitis virus posttranscriptional regulatory element (WPRE) and polyadenylation signal (pA), flanked by two asymmetrical homology arms (HAs (396bp and 420bp) (Figure 1B). Following editing with this gRNA/Cas9/AAV6 approach, successful HDR-mediated integration of the HDR cassette was assessed by flow cytometry examining GFP expression. Rates of HDR (GFP+) in wild-type CD3+ cells were 55.8 +/-1.6% (mean +/- SD n=3) (Figure 1C and Supplementary Figure 1B and 1C).

### Restoration of CTLA4 expression following correction of a point mutation

To demonstrate that we could use CRISPR/Cas9/AAV6 gene editing to correct CTLA4 insufficiency we first set out to repair the disease-causing c.370A>C (p.T124P) point mutation. Since gRNA 1 covers this site modification by one base was sufficient to retarget the guide to the c.370A>C mutation (gRNA 2: GATGTAGAGTCCCGGGTCCA). A second AAV6 HDR donor was also designed with a codon divergent CTLA4 exon 2 sequence (donor 2) to allow identification of HDR corrected alleles by sequencing (Supplementary Figure 1D).

Patient cells were then edited using gRNA 2/Cas9 RNP and HDR donor 2. Total CTLA4 median fluorescence intensity (MFI) increased following editing (from 149 +/- 1 (mean+/-SD) in unedited patient cells (n=3) to 167.7 +/- 3.8 following editing compared to 192.8 +/- 22.1 in healthy donor T cells (n=5)). After editing, the difference between healthy control and mutant CTLA4 MFI was no longer significant (p=0.036 prior to editing, p=0.071 post editing) (Figure 1D). Correction of the heterozygous mutation was confirmed following DNA extraction using in-out PCR and Sanger sequencing of the edited locus (Supplementary Figure 1E). Importantly, this guide (gRNA2) demonstrated specificity for only the mutated allele since no CTLA4 knockdown was observed in heathy cells treated with this guide (Figure 1E). These data demonstrate the feasibility of targeting patient mutations using mutation specific CRISPR guides and an AAV6 directed HDR repair approach.

### Assessment of universal gene editing strategies for correction of physiological CTLA4 expression in human T cells

Whilst promising, the above mutation-specific correction approach for CTLA4 insufficiency would not be feasible for clinical translation as over 50 distinct mutations have been described and new variants are being regularly discovered.^5^ We therefore set out to devise a universal editing strategy that could correct most disease-causing mutations.

**Figure 2:**
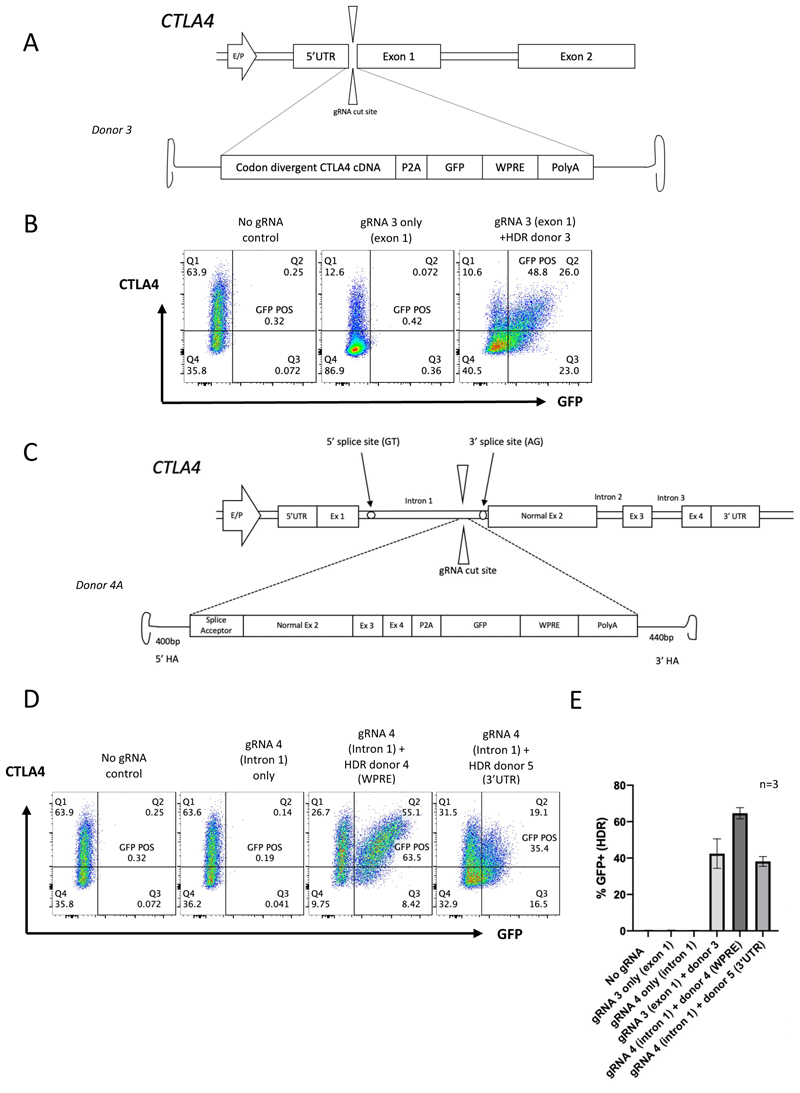
Universal editing strategies (A) Schematic representation of the editing strategy using gRNA 3 (exon 1) and donor 3 (HA-CTLA4-P2A-GFP-WPRE-HA). (B) Representative flow cytometry plots of the editing strategy shown in (A) demonstrating a non-edited control (left), gRNA only control with resulting knock down of CTLA4 (centre) and HDR mediated by the CTLA4 cDNA-P2A-GFP-WPRE AAV6 donor (48.8% GFP positive cells). (C) Schematic representation of the the intronic editing strategy (donor 4 HA-splice acceptor-CTLA4 exons 2, 3, 4-P2A-GFP-WPRE-HA) (D) Representative flow cytometry plots plots showing CTLA4 expression and GFP expression (HDR) in cells edited with the gRNA 3/Cas9 RNP alone (intron 1) (centre left), and then with transduction of donor 4 (WPRE) and donor 5 (3’UTR) (far right). (E) Mean HDR rate (n=3, percentage GFP+ in cells from separate healthy donors). Exon 1 approach (gRNA 3 + donor 3) mean=42.47% GFP+, SD 8.13, intronic WPRE donor (gRNA 4 + donor 4A) mean=64.63% GFP+, SD 3.06, Intron 3’UTR donor (gRNA 4, donor 4B) mean=38.13% GFP+, SD 2.70.

We therefore evaluated several universal editing strategies, first targeting early exon 1 of *CTLA4*. An AAV6 HDR template was designed with a wild-type CTLA4 cDNA in front of the P2A sequence, GFP reporter cassette and WPRE sequence flanked by two asymmetrical HAs (HDR donor 3) (Figure 2A). The target TGGCTTGCCTTGGATTTCAG (gRNA 3: UGGCUUGCCUUGGAUUUCAG) resulted in knock down of CTLA4 on assessment by flow cytometry (Figure 2B, middle plot) and produced on-target “indels” in >90% of CD4+ T cells when analyzed by inference of CRISPR edits (ICE) analysis (data not shown). This gRNA/AAV6 editing strategy was then tested in healthy human T cells. HDR was assessed by flow cytometry (GFP+) and showed average editing efficiencies (GFP+) of 42.5 +/- 8.1% (mean +/- SD, n=3), in healthy donor CD4+ T cells (Figure 2B, 2E).

In a second approach, we targeted the 3’ end of the first intron of *CTLA4*. This would enable correction of most disease-causing mutations but has the additional advantage of avoiding the introduction of indels in the coding region of the remaining healthy allele which could worsen disease in heterozygous disease settings. *In vitro* assessment demonstrated that using intron-targeting gRNAs, CTLA4 expression remained intact despite the creation of a dsDNA break (Supplementary Figure 2 A, B and C). On molecular assessment (inference of CRISPR edits (ICE)) analysis, one gRNA (gRNA 4: AGCUCCGGAACUAUAAUGAG) efficiently targeted the intron and produced indels in over 90% of cells while CTLA4 expression remained intact as measured by flow cytometry (Supplementary Figure 2A and B).

A new AAV6 HDR donor template was therefore designed incorporating an artificial splice acceptor (SA) sequence, followed by cDNA for exons 2, 3 and 4, P2A sequence, GFP reporter, WPRE and pA sequences flanked by two HAs (donor 4A) (Figure 2C). The SA sequence is required as the gRNA results in a dsDNA break prior to the endogenous splice acceptor thus allowing the artificial SA sequence in the HDR donor to exploit normal splicing of exon 1 to exon 2 of the repair donor following HDR. A further HDR donor template was also tested in which the WPRE-pA was replaced with the *CTLA4* 3’UTR, to allow comparison of the WPRE and the 3’UTR on the gene expression profile (donor 4B). Encouragingly, good editing efficiencies (64.6% GFP+ +/- 3.1% (mean +/- SD) (n=3)), were achieved using the intronic editing approach (Figures 2D and 2E), which were more efficient that the previous approach targeting exon 1(Figure 2E). When comparing the two HDR donors which differed by having a synthetic 3’UTR sequence (WPRE – donor 4A) or the CTLA4 3’UTR (donor 4B), the WPRE donor (donor 4A) reproducibly mediated higher editing efficiencies (Figures 2D and 2E). Confirmation of editing efficiency using gRNA 4 and donor 4A was performed using digital droplet PCR (ddPCR) with probes targeting the edited sequence, demonstrating targeted integration of ≥40% (Table, Supplementary Figure 2D).

The intronic gRNA with donor 4A (WPRE) was therefore selected for further validation.

Predicted on-target and off-target activities of gRNAs were initially assessed using *in silico* design tools (see methods). However, following demonstration of the superiority of the intronic editing strategy with gRNA 4, genome wide, off-target cleavage activities were formally assessed using capture of a short double-stranded oligonucleotide at double strand breaks (DSBs) through GUIDE-seq (genome wide, unbiased identification of DSBs enabled by sequencing) (see methods). GUIDE-seq analysis demonstrated that gRNA 4 had no detectable off-target activity (Supplementary Figures 2E and 2F).

### CTLA4-mediated transendocytosis is normal in edited CD4+ T cells and Tregs

**Figure 3:**
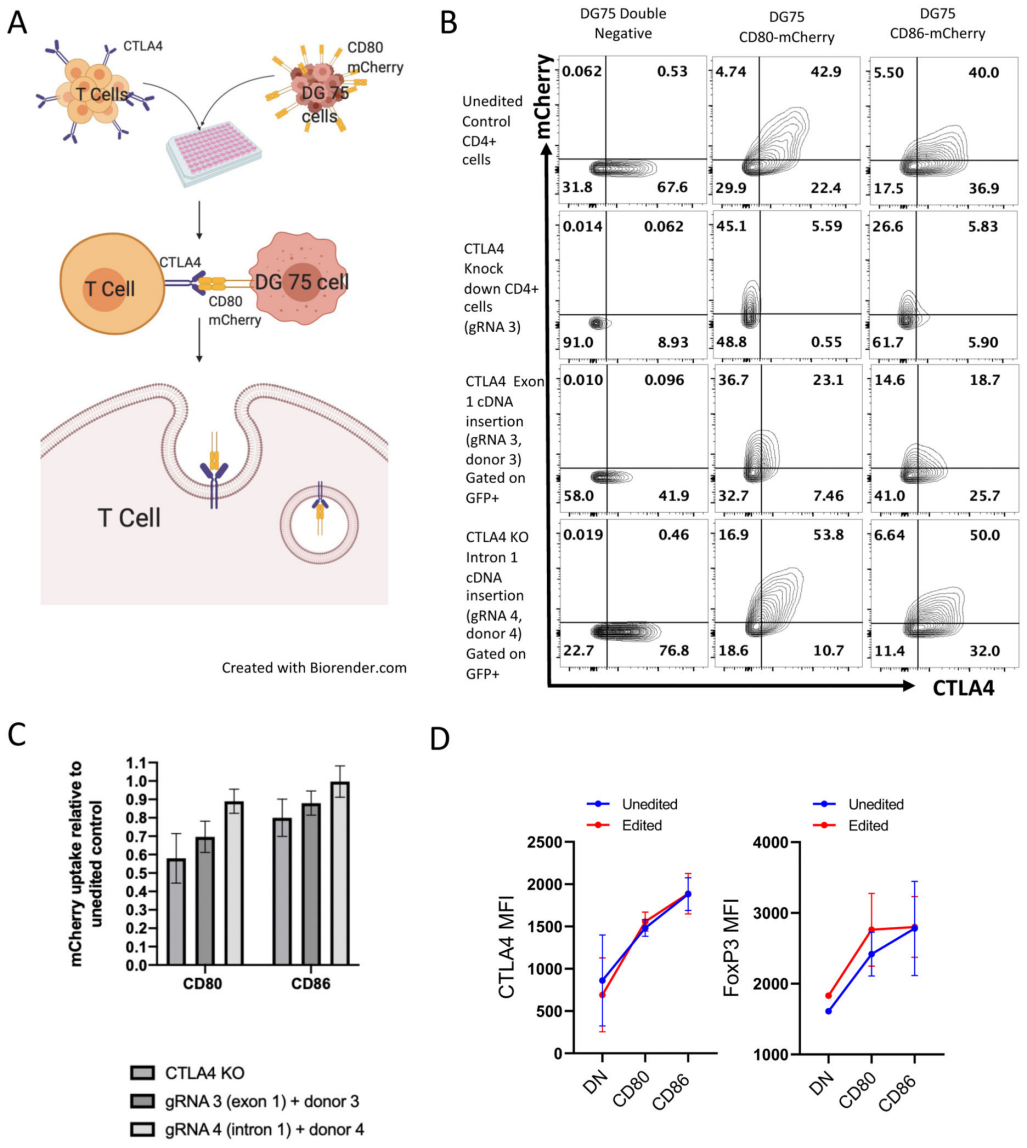
Functional characteristics of edited T cells (A) Schematic representation of transendocytosis assay. Cells (edited or unedited controls) are incubated in a 5:1 ratio with DG75 cells expressing either fluorescent labelled (mCherry) CD80 or CD86. Uptake of ligand can then be assessed by flow cytometry (mCherry uptake into T cells). (B) Representative FACS plots demonstrating TE of mCherry-bound CD80 and CD86 (top right quadrant each plot) in healthy control CD4+ T cells (top row), CD4+ cells that have undergone knock out of CTLA4 (upper middle row) and CD4+ cells that have undergone repair using the different editing strategies (gated on edited GFP+ cells). DG75 cells that do not express either ligand are used as a negative control (left column). (C) mCherry uptake relative to the unedited control with the different universal editing strategies in healthy CD4+ cells (CTLA4 KO mean = 0.69, SD = 0.155, N=3, gRNA 3 + donor 3 mean = 0.79, SD = 0.13, n=3, gRNA 4 +donor 4 mean = 0.94, SD = 0.08, n=3). (D) Graphs showing increase in CTLA4 and FOXP3 MFI in unedited cells (blue) and edited cells (red) when costimulated with CD80 and CD86.

We assessed the ability of our edited T cells to perform CTLA4-mediated transendocytosis (TE) using TE assays, whereby CTLA4 drives the capture of labelled ligands (CD80 and CD86) from donor B cells (Figure 3A).^11,34^ The ability of CD4+ T cells to perform CTLA4-mediated TE is monitored via transfer of mCherry-labelled CD80 and CD86 ligands from the surface of co-cultured B cells to the T cells, by flow cytometry (Figure 3A). Unedited healthy donor CD4+ cells were compared to cells that were edited with the universal editing approaches (donors 3 and 4A). Knock down of CTLA4 (91% using gRNA3 (exon 1)) without a repair donor almost entirely abolished TE (92% and 93% reduction in CD80-mCherry and CD86-mCherry uptake respectively (Upper right quadrant, second line, Figure 3B)). Importantly, TE was restored in cells now expressing CTLA4 particularly those edited with the intronic approach (gRNA 4), where donor 4A (WPRE) was the most successful in restoring ligand uptake equivalent to wild-type unedited cells (gating on edited GFP+ cells) (Figures 3B and 3C).

To demonstrate the intracellular transfer of CD80 and CD86 into CTLA4+ Tregs we amended our editing protocol to enable confocal microscopy to be performed on the cells of interest, using *in vitro* expanded Treg (Supplementary Figure 3A and methods). Using the intronic editing strategy (gRNA 4, donor 4A), HDR levels (determined by %GFP positivity) of up to 35% were achieved (Supplementary figure 3B). Confocal microscopy of the cells following 6-hour TE, demonstrated correct intracellular localization of CTLA4 itself as well as co-localization of CTLA4, CD80 and CD86 in GFP positive edited cells, which was indistinguishable from unedited cells (GFP-ve) in the same field. These data suggested TE of CD80 and CD86 in the edited Tregs was equivalent to that observed in unedited Tregs. (Supplementary figure 3C). Together these data demonstrated that our intronic editing approach produced functional CTLA4 that retains a similar ability to transendocytose CD80 and CD86 as seen in unedited healthy Tregs.

### CD4+ T cells with edited CTLA4 retain normal functional characteristics

To determine the functional characteristics of our edited T cells, we assessed the impact of gene editing on Treg survival. Since Treg require CD28 signalling for their homeostasis, we incubated edited and unedited Treg with DG75 B cells expressing either CD80, CD86 or no ligand. This also assessed the impact of CTLA4 expression since CTLA4 competes with CD28 for ligand binding in this system. Edited T cells were flow cytometrically sorted for GFP and compared with mock edited cells. After 5 days, both unedited and edited CD4+ T cells possessed a robust population of FoxP3+ Tregs following stimulation in the presence of CD80 or CD86, indicating that edited cells behaved indistinguishably from unedited cells (Supplementary figure 3D). In addition, we observed that in both unedited and edited cells CD86-CD28 costimulation enhanced the expression of CTLA4 compared to CD80-CD28 costimulation (Figure 3D) in line with previous observations^35^. Together, this data indicated that Treg homeostasis, which integrates both CD28 and CTLA-4 function was normal in our edited cells.

Next, we assessed the ability of edited T cells to produce cytokines in response to stimulation. Unedited and sorted edited CD4+ T cells were rested for 72 hours and then stimulated for 4 hours with phorbol myristate acetate (PMA)/ionomycin. Cells were then fixed, permeabilized and intracellular cytokine staining performed. This revealed that both Interferon gamma and IL-17 production was similar in edited CD4+ cells compared to unedited controls (Supplementary Figures 4A, B) again supporting the observation that gene editing does not affect the functional characteristics of T cells *in vitro*.

### Universal intronic gene editing strategy restores CTLA4 expression and function in CD4+ patient T cells

**Figure 4:**
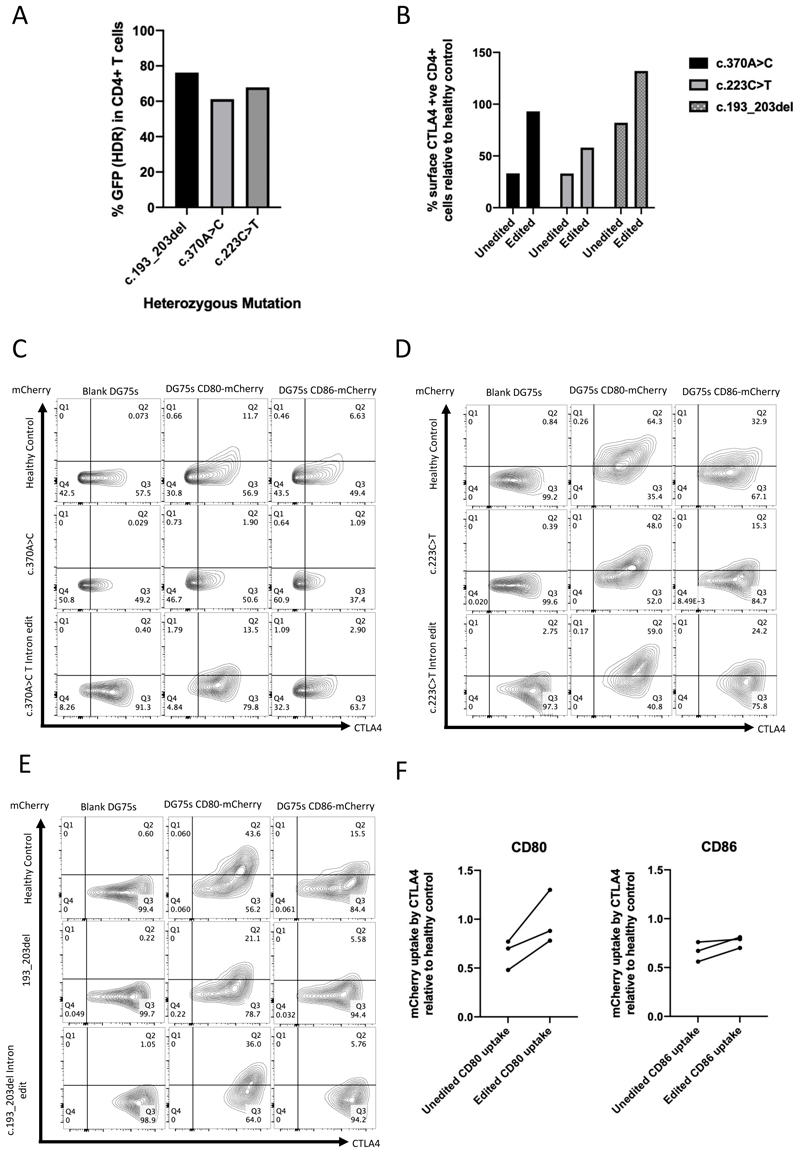
Restoration of CTLA4 expression and function in CD4+ cells from patients with CTLA4 haploinsufficiency (A) HDR rates (% cells GFP+) in edited CD4+ cells from patients with CTLA4 haploinsufficiency resulting from three different mutations. (B) Graph showing restoration of surface CTLA4 in heterozygote mutant CD4+ cells following editing with gRNA 4 and HDR donor 4. %CTLA4 positive relative to a healthy control assessed at the same time are shown. GFP+ edited cells are compared to mock edited cells. (C, D, E) Overnight TE assays gated on CD3+ CD4+ FOXP3+ cells in healthy control (top rows), patient cells with three different mutations (middle rows) and patient cells corrected with the intronic editing strategy (bottom rows). (F) Graph showing the increase in ligand acquisition (% CD4+ FOXP3+ T cells mCherry positive) in patient cells after editing relative to healthy control.

Having established our gene editing strategy in healthy control cells we proceeded to test this system in patient-derived material. We obtained cells from three patients with CTLA4 insufficiency resulting from three different heterozygous mutations (c.370A>C, c.193_203del and c.223C>T). Gene editing of CTLA4 insufficient CD4+ T cells was performed using the universal intronic approach (HDR donor template 4A – WPRE) and was assessed by flow cytometry for editing efficiency and restoration of CTLA4 expression. Similar editing efficiencies to those achieved previously with healthy donor T cells were obtained in patient samples, with HDR rates (determined by %GFP+) >60% in all patient samples tested (Figure 4A). Further, editing restored surface CTLA4 expression levels in CD4+ T cells to levels similar to healthy controls (Figure 4B).

We then tested the ability of edited cells to perform TE, using Tregs due to higher constitutive expression of CTLA4 and higher efficiency of TE in these cells. Following overnight TE assay, cells were fixed and permeabilized and stained for total CTLA4 and the transcription factor FOXP3 allowing TE to be examined in the Treg fraction. In all three patient samples a reduction in TE compared to the healthy control was noted prior to gene editing (Figures 4C, 4D and 4E second rows) however, there was not a complete absence of TE due to the remaining functional allele in these patients. Nonetheless, following intronic editing (gRNA 4, donor 4A), TE was restored to healthy donor levels (Figures 4B, 4C, 4D, 4E and 4F), demonstrating that our universal gene editing approach restored CTLA4 expression and function in patient-derived T cells with three different heterozygous mutations in *CTLA4*.

Whilst protein expression profiles between WT unedited and cells edited with gRNA 4 and HDR donor 4A were similar to each other, we also assessed the effects of gene editing on transcription. Cells were rested for 72 hours following editing (or mock editing) and then restimulated for 48 hours with CD3/CD28 beads. mRNA was extracted and quantified by qPCR. Reassuringly mRNA expression was similar between unedited and edited healthy control CD4+ T cells (Supplementary Figure 4C). The same experiment was performed on unedited and edited CD4+ T cells from patients with two different heterozygous mutations in *CTLA4* that are known to reduce CTLA4 protein expression (c.370A>C and c.223C>T). Following editing with gRNA 4, donor 4A, CTLA4 mRNA expression increased compared to the unedited heterozygous cells (Supplementary Figure 4D) supporting the flow cytometric results obtained for the same cells described earlier (Figure 3B).

### Edited cells retain normal kinetics of CTLA4 surface expression in the resting and activated states

**Figure 5:**
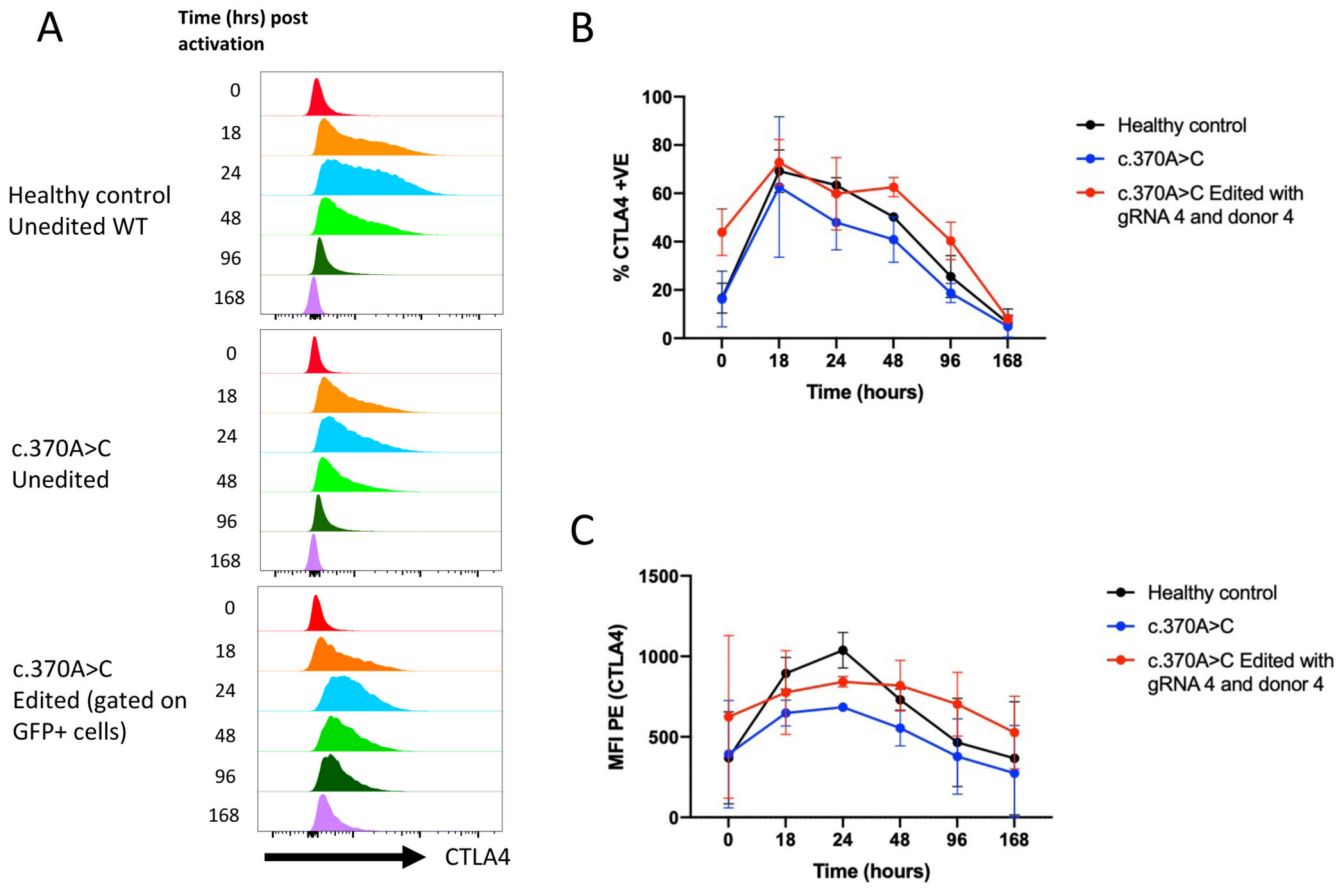
Kinetics of CTLA4 surface expression in resting and activated states (A) Representative time course of CTLA4 expression (MFI histogram) on healthy control CD4+ cells (top), c.371A>C heterozygous mutant cells (middle) and c.371A>C heterozygous mutant cells edited with gRNA 4/Cas9 RNP and donor 4. (B) Percentage and MFI (C) of CTLA4 surface expression over time (n=3 for all conditions).

Given that CTLA4 expression is normally tightly controlled we wished to establish that CTLA4 expression post editing had the same profile as healthy unedited cells. The surface expression of CTLA4 (staining performed at 37°C to maximize cycling) on unedited and intronic edited (donor 4A) T cells was therefore monitored over a 7-day period. 4 days post editing, cells were re-activated with CD3/CD28 beads and surface expression of CTLA4 was analyzed by flow cytometry at 18-, 24-, 48-, 96- and 168-hours. CTLA4 expression in healthy control T cells peaked at 18-24 hours and then gradually returned to baseline by 168 hours (Figures 5A). Importantly, the expression kinetics of CTLA4 haploinsufficient cells edited with gRNA 4 and donor 4 (WPRE) mimicked those of healthy unedited cells and exhibited consistently higher median fluorescent intensity (MFI) than unedited patient cells (Figures 5B and 5C). This demonstrated the ability to preserve the normal expression patterns of CTLA4, which is one of the key advantages of using the endogenous CTLA4 promoter via gene editing over simple lentiviral gene replacement approaches for CTLA4 insufficiency.

For a point of comparison, we designed a lentiviral vector encoding *CTLA4* cDNA followed by a P2A-GFP sequence under the influence of a phosphoglycerate kinase (PGK) promoter (Supplementary Figure 5A). Healthy human T cells were transduced (at 80-90% efficiency, vector copy number 3.4) with this lentiviral vector (Supplementary Figure 5A). Overnight TE assays demonstrated increased TE of ligand compared to wild type CD4+ T cells and edited CD4+ T cells (Supplementary Figure 5B). The kinetics of CTLA4 surface expression in healthy and patient cells transduced with this vector were assessed and lentivirally transduced cells were observed to have higher expression of CTLA4 in both the resting and activated state (Supplementary Figures 5C and 5D). Together, the above data demonstrate that our intronic editing approach with gRNA 4 and HDR donor 4 produces CTLA4 that faithfully retains the expression kinetics of healthy T cells, providing evidence that the endogenous gene control machinery remains intact following gene editing.

### Assessment of T cell gene therapy for CTLA4 insufficiency in vivo using a murine model

The critical regulatory role of CTLA4 was first identified in CTLA4 knockout mice (CTLA4^-/-^) which exhibit a lethal lymphoproliferative syndrome with multi-organ lymphocytic infiltration.^36,37^ Since CTLA4 can regulate T cell responses in a cell-extrinsic manner (consistent with its role on regulatory T cells), the presence of CTLA4-sufficient T cells, in mixed bone-marrow chimeric mice or in adoptive co-transfer models, can correct the disease phenotype.^38^ We therefore devised a murine approach to test whether gene edited T cells could survive *in vivo* and control the disease associated with CTLA4-deficiency. Since the *CTLA4* gene is disrupted in CTLA4^-/-^ mice by mutations introduced in exon 2,^36^ a similar editing strategy targeting the first intron (of murine *CTLA4*) was used. Murine CTLA4^-/-^ T cells were edited (gRNA 5, HDR donor 5) or mock edited and then adoptively transferred into adult Rag2^-/-^ mice without conditioning. We have previously demonstrated that co-transfer of CTLA4-sufficient Treg can control the disease induced by CTLA4-deficient T cells in this system^39^, thus, sufficient restoration of CTLA4 expression by gene editing would be predicted to protect from lymphoproliferative disease.

**Figure 6:**
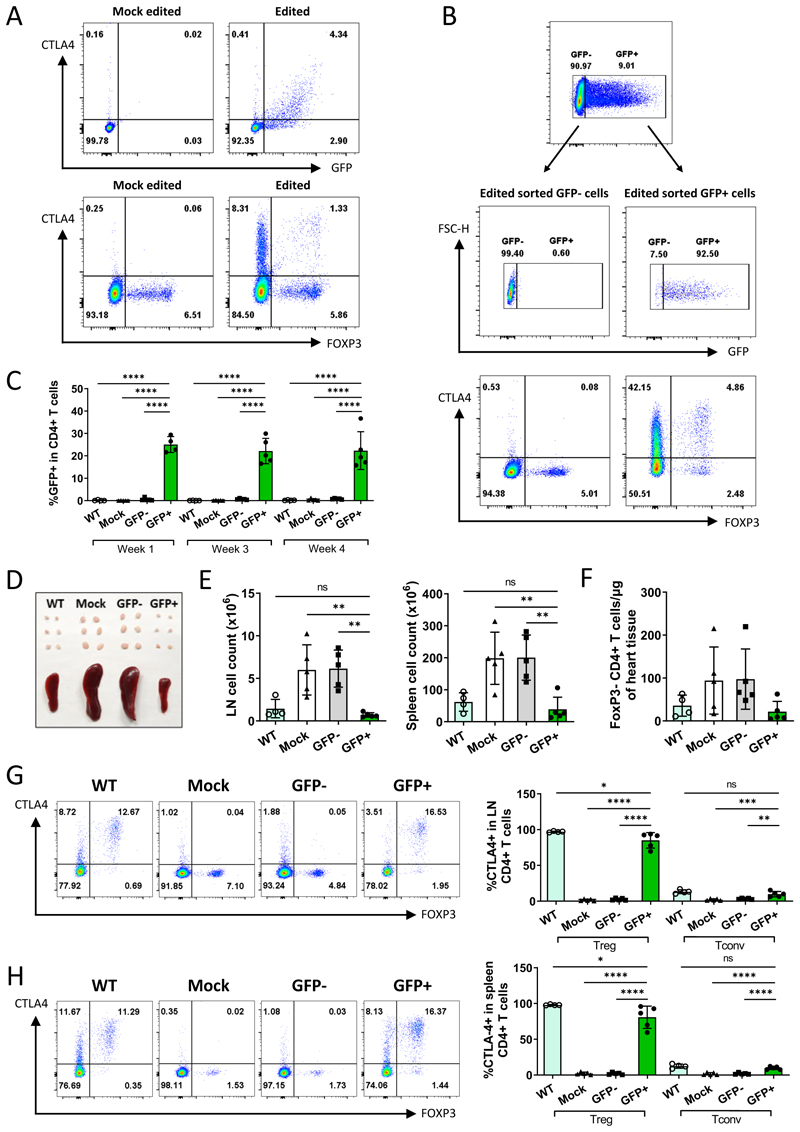
Assessment of T cell GT for CTLA4 insufficiency using an *in vivo* murine model (A) FACS plots demonstrating typical editing efficiencies achieved in murine CTLA4^-/-^ T cells (GFP+ cells, upper plots) with restoration of CTLA4 expression in both the FOXP3+ and FOXP3- compartments (lower plots). (B) FACS plots post sort demonstrating % GFP+ in the sorted edited cells (upper plots) and CTLA4 expression in FOXP3- and FOXP3+ cells in the two sorted populations (GFP- left lower plot, GFP+ left upper plot).(C) Serial tail vein bleeds demonstrating persistence and stability of the GFP+ population after adoptive transfer. (D) Lymph node and spleen size in mice that received wild type T cells (left) mock edited and GFP-cells (middle) and GFP+ enriched edited cells (right). (E) Lymph node (left) and spleen (right) cell counts in mice that received wild-type T cells, mock edited, GFP-and GFP+ T cells. (F) Number of Tconv per μg of heart tissue in mice that received WT, mock edited, GFP- and GFP+ T cells. (G) Representative FACS plots (left) and collated data (right) showing CTLA4 expression in lymph node Treg and Tconv cells. (H) Representative FACS plots (left) and collated data (right) showing CTLA4 expression in spleen Treg and Tconv cells. Data collated from 2 independent experiments; n=4-5. One-way ANOVA; mean ± SD are shown; ****, p < 0.0001; ***, p < 0.001; **, p < 0.01; *, p < 0.05; ns, not significant.

A gRNA was selected that causes a dsDNA break in the 3’ end of the first intron of murine *CTLA4*. An AAV6 HDR repair template was designed, replicating the architecture of the human template but containing the murine genomic sequence (donor 5) (Supplementary figure 6A). Editing efficiencies were lower than in human T cells however cycling CTLA4 molecules could only be detected in the GFP+ fraction of the CTLA4^-/-^ cells, confirming successful gene expression (Figure 6A, upper panel). Likewise, intracellular staining revealed restoration of CTLA4 expression in both Treg (Foxp3+) and Tconv (Foxp3-) compartments (Figure 6A, lower panel) as expected. The lower editing efficiencies were mitigated by fluorescence-activated cell sorting (FACS) on GFP positivity to produce a cellular product with a high proportion of edited cells (Figure 6B). The sorted GFP negative cells were used as a control population that were matched for activation/editing conditions having derived from the same wells as the GFP+ cells. Additional controls included mock-edited CTLA4^-/-^ cells and unmanipulated wild-type T cells. 6x10^5^ edited or control cells were injected intravenously into Rag2^-/-^ mice (Protocol schematic in supplementary figure 6B). Tail vein bleeds were performed 1, 3 and 4 weeks post adoptive transfer. In the mice which received the GFP+ edited cells a stable population of GFP+ cells were detectable at all timepoints demonstrating *in vivo* persistence as well as genetic stability (Figure 6C).

All mice were sacrificed 4 weeks after cell transfer. To assess lymphoproliferation, the cellularity of peripheral lymph nodes and spleen weight were analyzed. When peripheral lymph nodes and spleens from all treatment groups were compared, lymphadenopathy and splenomegaly could be observed in mice that had received mock-edited and edited GFP- T cells (edited, but without repair) while lymph nodes and spleens from mice treated with edited GFP+ T cells did not differ from those found in the recipients of WT T cells (Figure 6D). Furthermore, lymph nodes and spleens from recipients of edited GFP+ and WT T cells displayed equal cellularity whereas lymph nodes and spleens from mice treated with mock-edited and GFP- T cells contained significantly greater cell numbers (Figure 6E). To assess lymphocytic organ infiltration, cardiac tissue was analysed: only mice from groups that had received mock-edited or GFP- T cells showed elevated cardiac tissue infiltration while Tconv numbers in mice treated with edited GFP+ cells did not exceed those seen in WT controls (Figure 6F). Collectively, these findings indicated that the lymphoproliferative disease that occurred in recipients of CTLA4^-/-^ cells was being controlled in mice that received edited GFP+ cells. Subsequent assessment of CTLA4 levels in cells from the lymph nodes (Figure 6G) and spleens (Figure 6H) of recipient mice revealed that CTLA4 was expressed in over 70% of lymph node Treg and over 60% of splenic Treg and in 6-15% of Tconv in mice that had received edited GFP+ T cells. Indeed, CTLA4 expression in edited GFP+ Treg was only marginally lower than that seen in wild-type Treg, while expression in edited Tconv was equivalent to wild-type levels.

Together these data demonstrated that CTLA4 edited T cells survived *in vivo*, expressed CTLA4 and were able to control the clinical phenotype of CTLA4 insufficiency, providing a powerful proof-of-principle for our T cell GT approach.

## Discussion

Here, we demonstrate that gene editing approaches for CTLA4 insufficiency result in correction of the immunological defects and provide a universal editing strategy, which is attractive for clinical translation. An autologous T cell GT may improve the clinical phenotype, whilst abrogating many of the immunological complications of alloHSCT as well as removing the need to find a suitably matched donor. Targeting the first intron was superior in terms of editing efficiency with the key advantage of avoiding the introduction of new indels which could potentially worsen disease, by targeting the remaining healthy allele. Several studies have demonstrated that gene expression can be enhanced in mammalian cells by the inclusion of an intron, such as when correcting *CYBB* gene mutations in chronic granulomatous disease (CGD) and when editing the CD40 ligand gene.^39–44^ However, our intronic editing approach targets the 3’ end of the first intron of *CTLA4* thus preserving the majority of the first intronic sequence.

Most gene therapy approaches for IEIs modify hematopoietic stem cells (HSCs). However, for disorders mediated primarily through the lymphoid compartment there are clear advantages of a T cell GT approach most notably the requirement for less intensive conditioning and higher editing efficiencies.^32^ T cell GT for IEIs could benefit from the rapidly expanding infrastructure to manufacture genetically engineered T cell products.^45^ Correction of HSCs enables long term correction due to modification of a self-renewing population of cells^46^, however, increasing data suggests that genetically engineered CAR-T cells can also persist long term if sufficient numbers of central and effector memory T cells are modified and transfered.^47^ Clinical proof-of-principle of T cell gene therapy for IEIs already exists from the early retroviral T cell gene therapy trials for ADA-SCID which demonstrated persistence of gene marking 10 years after patients received gene-modified T cells.^48^

CTLA4 insufficiency is a complex disorder, and the pathology may not be strictly confined to the CD4 T cell compartment. Abnormalities in other immune cell lineages such as natural killer cells and B cells have been observed in CTLA4 insufficiency.^1,3,49^ It is therefore important to extend this work to the correction of autologous HSCs and to compare an HSC GT approach to T cell GT. Although exploration of HSC GT for CTLA4 insufficiency needs to be explored, we would hope to position a T cell GT strategy ahead of HSC editing due to the reasons outlined in the introduction including, less intense conditioning, use of non-mobilized apheresis, reduced risk of mutagenesis and higher editing efficiencies. The cell-extrinsic action of CTLA4 makes a T cell GT particularly attractive for the disorder.

It is currently not known what degree of correction is required to ameliorate the clinical phenotype in CTLA4 haploinsufficiency. In humans, alloHSCT can be curative although in the cases reported the majority have 100% donor chimerism.^5,8^ However, from murine experiments it is clear that 50:50 chimeras or less can correct disease in CTLA4^-/-^ mice ^37,50^ and that in adoptive transfer models, a single injection of CTLA4-sufficient Treg can prevent disease caused by CTLA4-deficient bone marrow. ^39,36,37,50^ The limitation of incomplete correction could be mitigated in a clinical product by selecting cells using a reporter gene as shown in our *in vivo* experiments. In our editing construct we included a GFP tag which could be easily substituted for a clinically appropriate reporter such as truncated nerve growth factor receptor (NGFR) to enable a cell product that contained close to 100% edited cells.^51^

Our data provide proof-of-concept that gene editing can restore CTLA4 function in human T cells demonstrating the potential of this approach to treat CTLA4 haploinsufficiency. Targeting the first intron of *CTLA4* was the most effective and widely applicable strategy and the absence of detectable off-target edits from the gRNA used suggests that the safety of this approach is promising. Further work should assess this editing approach in HSCs. A similar approach could be used in other IEIs that are caused by multiple heterozygous mutations.

## Materials and Methods

### gRNA design and validation

CRISPR guide RNAs (gRNAs) were designed using the Benchling online tool (https://www.benchling.com/crispr/). NGG PAM sequences were identified, and gRNAs assessed *in silico* for on-target and off-target activity. The top three scoring gRNAs were ordered from Synthego (Synthego, USA) and assessed *in vitro*. 72 hours following nucleofection, DNA was extracted using QuickExtract™ (Cambio). Primers were designed and PCR performed on extracted DNA to create amplicons 800bp in length that included the site of the predicted dsDNA break. Amplicons were sent for sanger Sequencing. Sequencing results were then analyzed using the Synthego ICE software (ice.synthego.com). The gRNA that caused the highest percentage of indels (>85%) was selected for the editing approach. The gRNA selected for each approach is detailed in table 1.

### AAV6 donor template manufacture and production

Donor templates were designed using Snapgene (Snapgene, USA) software and incorporated asymmetrical homology arms 396bp and 420bp in length which flanked the sequence to be inserted at the site of the dsDNA break. Sequences for the insert were manufactured by Geneart™ (Thermofisher, USA). This insert was then cloned into a AAV6 vector. AAV vectors were produced with a double transfection method that introduces an ITR-containing transfer plasmid and a single helper plasmid, pDGM6 (obtained from the Russell laboratory at the University of Washington with permission) which contains the AAV2 rep and AAV6 cap proteins.^52^ Vector production took place in HEK293T cells seeded at 15x10^6^ in Complete Dulbecco’s Modified Eagle Medium (DMEM) media (Life Tech, USA) in 15x15cm dishes. 24μg of pDGM6 (per plate) and 12μg of ITR-containing plasmid was used to transfect cells and branched polyethylenimine added at a 4:1 ratio to DNA. 48 hours later, supernatant was harvested, treated with ammonium sulphate (Sigma Aldrich, UK) (31.3g per 100ml supernatant), pelleted (centrifuge at 8300xg for 30 minutes) and re-suspended in 10ml total volume of 1xTagment DNA (TD) buffer (diluted from 5xTD: 5xPBS, 5mM MgCl_2_, 12.5mM KCl). This solution was then treated with 50U/ml Benzonase (Sigma Aldrich, UK) and incubated at 37°C for 30 minutes and stored at 4°C before purification. Simultaneously, cells were harvested using cell scrapers, pelleted (centrifuge 1400xg for 10 minutes), washed and resuspended in 10ml 1xTD buffer. Three freeze-thaw cycles were performed, 0.5% deoxycholic acid sodium salt (VWR, USA) and benzonase 50U/ml added and the solution incubated for 30 minutes at 37°C. The lysate was pelleted (4000xg for 30minutes at 18°C) and supernatant removed and stored at 4°C prior to purification. The two solutions were combined and AAV6 vectors purified by Iodixanol density gradient and ultra-centrifugation at 40,000rpm (273,799xg) for 2 hours at 18°C. AAV6 particles were extracted using a needle and syringe between the 40% and 60% gradient interface and dialyzed 3 times in 1 x PBS (ThermoFisher, USA) with 5% sorbitol (sigma-Aldrich, UK) in the third step using 10K MWCO Slide-a-Lyzer dialysis cassettes (ThermoFisher, USA). Titration was performed using Quick Titre AAV Quantification Kit (Cell Biolabs, USA) prior to aliquoting and storage at -80°C before use.

### Cell Isolation and Culture

Peripheral blood mononuclear cells (PBMCs) were isolated from patients with CTLA4-haploinsufficiency and healthy controls and diluted 1:2 with PBS prior to layering over Ficoll (Sigma Aldrich) and centrifugation (1060 x g for 22 minutes). The PBMC layer was collected, washed and cells frozen in a solution containing FBS and 20% dimethylsulfoxide (DMSO) and stored in liquid nitrogen until use.

After thawing, T cells were isolated by MACS using the CD4+ T Cell Isolation Kit (Miltenyi Biotec). CD4 selected human cells were cultured in TexMACS Media (Miltenyi Biotec, 130-097-196) supplemented with 1% Penicillin/Streptomycin (100 U/ml; GIBCO, 15070), human IL-2 (Roche 11147528001) 10U/ml (1000U/ml for T_regs_), human IL-7 (BD, 554608) 5ng/ml and human IL-15 (BD, G243-886) 5ng/ml T cells were activated via CD3/CD28 stimulation by using T cell Transact (Miltenyi Biotec 130-111-160) 1:100 titer.

### Treg Isolation and Expansion

For Treg isolation, CD4+ T cells were enriched by addition of RosetteSep™ Human CD4+ T Cell Enrichment Cocktail (Stemcell Technologies) to leukocyte cones diluted 1:5 with phosphate buffered saline (PBS), prior to as per manufacturer’s instructions. Blood was layered over Ficoll-Paque PLUS (GE Healthcare) and centrifuged at 1200g for 25 minutes with slow acceleration and no brake. The CD4 enriched layer was collected, washed twice in PBS, before isolation by immunomagnetic positive selection using human CD25 MicroBeads II (Miltenyi Biotec) according to the manufacturer’s instructions. Enriched CD4+CD25+ cells were stained using an antibody cocktail (Anti-CD4 (RPA-T4), anti-CD25 (3G10) and anti-CD127 (A019D5)) and FACSAria sorting was used to sort CD4+CD25+CD127lo Tregs. Sorted Tregs were expanded by plating at a 1:1 ratio with irradiated DG75 cell lines stably expressing CD86 co-stimulatory ligand in the presence of 1000 IU/ml IL2 (PeproTech) and 1μg/ml of anti-human-CD3 (OKT3, Biolegend), with IL2 replenished every 2-3 days.

### Treg Stimulation Assay

DG75 cell lines underwent CRISPR-Cas9 HDR editing to knock-out endogenous CD80 and CD86 co-stimulatory ligands (DG75-DN). Edited DG75 were transduced to stably express CD80-GFP or CD86-GFP. Stably transduced DG75-DN, -CD80-GFP or CD86-GFP were irradiated at 7500rads, and incubated with edited or unedited T cells at a 1:1 ratio in the presence of 1000 IU/ml IL2 (PeproTech) and 1μg/ml of anti-human-CD3 (OKT3, Biolegend) for 5 days, with IL2 replenished every 2-3 days. At Day 5 post-stimulation, T cells were fixed and permeabilized using the FoxP3 staining kit (eBioscience) and stained with anti-CTLA4-PE (clone BNI3; BD Biosciences) and anti-FoxP3 Pe-Cy7 (236A/E7; Thermo Fisher Scientific) before flow-cytometric analysis using an LSR Fortessa (BD Biosciences).

### Electroporation and Transduction

HiFi Cas9 (Integrated DNA technologies (IDT, USA)) and gRNA were mixed at a 1:3 molar ratio and incubated at 25°C for 30 minutes to form RNPs. A Lonza Nucleofector 4D was used for nucleofection (programme EO-115) with a P3 Primary Cell 4D-Nucleofector Kit (Lonza, V4XP-3032). 1x10^6^ CD4+ or T_reg_ cells per reaction were washed in PBS and resuspended in 15μl/per reaction of P3 nucleofector solution. Cells were mixed 1:1 with RNP solution (30μl total volume) and transferred to the nucleofector strip. Immediately following nucleofection, 80μl of warmed TexMACs media was added and cells transferred to a 24 well plate containing 920μl of warmed TexMACS Media (Miltenyi Biotec, 130-097-196) supplemented with 1% Penicillin/Streptomycin (100 U/ml; GIBCO, 15070), human IL-2 (Roche 11147528001) 10U/ml (100U/ml for T_regs_), human IL-7 (BD, 554608) 5ng/ml and human IL-15 (BD, G243-886) 5ng/ml for CD4+ cells. For isolated T_regs_ IL-2 (100units/ml) and aCD3 (100ng/ml) were used. AAV6 was added at 13,000 MOI (vector genomes/cell). After 24 hours cell density was adjusted to 0.5x10^6^/ml. Cells were phenotyped >48 hours post editing by flow cytometry.

### Transendocytosis (TE) assay

Prior to incubation, T cells were labelled with CellTrace Violet labelling kit (ThermoFisher Scientific C34571). Control or edited CD4+ T cells (or T_regs_) were incubated with ligand donor cells (DG-75 B cells) expressing CD80 or CD86 molecules C-terminally tagged with mCherry. Donor and recipient cells were plated at a 5:1 ratio (donor:recipient) in 96-well round bottom plates in TexMACS Media (supplemented with cytokines as detailed previously) and left in an incubator at 37°C overnight. Cells were stained as detailed below and analyzed by flow cytometry. TE uptake was determined by dividing the MFI of mCherry in the upper two quadrants of the experimental condition cells against the same MFI of the healthy control for that particular experiment

### Flow Cytometry

Flow cytometric analysis was performed on an LSR Fortessa (BD Biosciences) and data analyzed using FlowJo Version 10.7.0 (Treestar). The following anti-human antibodies were used: anti-CD3-PE-Cy7 (BD Biosciences, clone HIT3a), anti-CD4-V500 (BD Biosciences, clone RPA-T4), anti-CD25-APC (Biolegend, clone BC96), anti-CD152-PE (CTLA4) (BD Biosciences, clone BNI3), LIVE/DEAD™ Fixable Near-IR dead cell stain kit (ThermoFisher L10119). Intracellular staining was performed using the eBioscience™ Foxp3/Transcription factor staining buffer set (ThermoFisher 00-5523-00) and anti-FOXP3-APC (ThermoFisher, clone 236A/E7) and anti-GFP-FITC (Rockland, 600-402-215).

The following anti-mouse antibodies were used: anti-CD3-BV421 (BD Biosciences, clone 17A2), anti-CD4-BUV737 (BD Biosciences, clone GK1.5), anti-CD4-PerCP-Cy5.5 (BD Biosciences, clone RM4-5), anti-CD152-PE (CTLA-4) (BD Biosciences, clone UC10-4F10-11), anti-FOXP3-APC (ThermoFisher, FJK-16s), Fixable Viability Dye eFluor™ 780 (ThermoFisher).

### Imaging

DG75 expressing mCherry-tagged CD80 or CD86 and Treg were seeded in a 2:1 ratio into 96 well U-bottom plates for 6 hours, washed with ice-cold PBS, resuspended in ice-cold 4 % PFA (ThermoFisher 28908) in PBS, transferred into 0.01% Poly-L-Lysine coated wells of an imaging plate (Greiner 655866, Sigma Aldrich A-005-C) and spun at 500 g for 20 min. PFA was quenched with 50 mM NH4Cl in PBS. Cells were then washed three times with PBS, permeabilized with 0.1% Saponin in PBS and stained with anti-CTLA4 clone C19 (Santa Cruz sc-1628) in 0.1% Saponin supplemented with 5% BSA (staining buffer) at 4°C overnight. After three Saponin washes, cells were incubated with donkey-anti-goat secondary AlexaFluor647 antibody (4μg/ml Thermo Fisher A-21447) and DAPI (2μg/ml, D9542) in staining buffer for 40 min at room temperature, washed three times in Saponin, two times in PBS and two times in miliQ water and mounted in Mowiol mounting medium with 2.5% DABCO. Imaging was performed on a Nikon Eclipse Ti confocal inverted laser scanning microscope equipped with a 60X oil-immersion objective (NA 1.4).

### Assessment of Nuclease Specificity

Small double-stranded oligonucleotides (dsODN) were obtained from Creative Biogene (NY, USA). Healthy CD4+ cells were isolated, activated and cultured as per previously described protocols. GUIDE-seq was performed on cells nucleofected with gRNA 4 (AGCUCCGGAACUAUAAUGAG) only. Editing was performed as per the previously described editing protocol except that 2μl 100nM per reaction of dsODN was added to the RNP solution at the end of the 30-minute incubation. 72 hours post editing genomic DNA was extracted using a QIAGEN Blood & Cell Culture DNA MiniKit (Qiagen, 13323) as per manufacturer’s instructions. DNA was suspended in 1ml/condition of 1xTris-EDTA (TE) buffer, frozen at -20°C and then shipped on dry ice to Creative Biogene who performed amplification and next-generation sequencing and analysis.

### Assessment of edited efficiency by ddPCR

In/out and ddPCR - Primers for detection of donors 4A at the *CTLA4* locus were designed using the NIH Primer-BLAST tool (Fwd: ATTGGATCATGGGGGACTCA; Rev: GCACGGTTCTGGATCAATTACA). For ddPCR, the same primers were used with the addition of a probe CTGGCCAGCAGCCGAGGC (5’6-FAM, Internal ZEN and 3’ Iowa Black FQ, IDT). The genomic reference amplicon primers targeted albumin (Fwd GCTGTCATCTCTTGTGGGCTG, Rev CACAAATTTGGAAACAGAACAGGCATT, amplicon length 1035bp) and probe CCTGTCATGCCCACACAAATCTCTCC (5’HEX, Internal ZEN and 3’ Iowa Black FQ, IDT). Droplets were generated and analyzed according to the manufacturer’s instructions (QX200 system, Bio-Rad). The cycling conditions were (95°C 10mins initiation, 50x (94°C 1min, 60°C 30s, 72°C 6min) 98°C 10mins, store 12 °C).

### Determination of mRNA levels by RT-PCR

Total RNA was extracted from cells using RNeasy miniprep kit (Qiagen) as per manufacturer’s instructions. RNA was converted to complementary DNA using LunaScript RT SuperMix Kit (New England Biolabs) as per manufacturer’s instructions. Relative expression levels of CTLA4 were measured by qPCR using CFX96 Touch Real-Time PCR Detection System (BioRad), TaqMan Fast Universal PCR Master Mix (2X) No AmpErase UNG (Applied Biosystems) and TaqMan FAM-labelled probes (ThermoFisher Scientific: CTLA4 Hs00175480_m1, GAPDH: Hs02786624_g1). Fold changes were calculated using the ΔΔCt method and results were normalized to the levels of GAPDH.

### Ethics approval for collection and use of human samples

Blood samples and biopsies were obtained with ethical approval (National Research ethics numbers 08/H0720/46, 99/095 and 02/208) and informed consent from all subjects in accordance with the Declaration of Helsinki.

### Mice

Rag2^-/-^ mice were purchased from Taconic Biosciences. CTLA4^-/-^ mice were a gift from A. Sharpe (Harvard, Boston, MA). Mice were housed in individually ventilated cages with environmental enrichment in a humidity and temperature-controlled environment with a 14-hour light, 10-hour dark cycle at the University College London Biological Services Unit. Experiments were performed in accordance with Home Office project and personal licenses with approval from University College London Animal Welfare Ethical Review Body. All injections were carried out in the afternoon, in the absence of anesthesia and analgesia, and mice were returned immediately to the home cage following the procedures. The welfare of adoptively transferred animals was monitored at least every 2 to 3 days. There was no blinding. Co-housed littermates were randomized to treatment groups such that treatment groups were spread across cages.

### In vivo experiments

Cells were isolated from lymph nodes (LN) of 16-20 day old male or female CTLA4^-/-^ mice and a negative CD4+ selection performed (MACS) (Miltenyi Biotech 130-104-454). Cells were re-suspended in Roswell Park Memorial Institute (RPMI) (Gibco 31870-025) medium supplemented with 10% fetal calf serum, 1% penicillin/streptomycin and 1% L-glutamine (Gibco) and IL-2 (Roche 11147528001) 30U/ml. Cells were stimulated for 24 hours with CD3/CD28 beads (Dynabeads^tm^ Gibco, 11452D). Beads were removed and cells edited using gRNA GGUCUUGGAAACUAAGCCUG Cas9 RNPs with a Lonza Nucleofector 4D (programme DN100) and P3 Primary Cell 4D-Nucleofector Kit (Lonza, V4XP-3032) before being immediately transduced with AAV HDR donor 5. Cells were rested for 48 hours and then sorted for GFP expression using a FACSAria Fusion (BD Biosciences). 6x10^5^ cells (GFP+, GFP- or mock edited) were then injected intravenously into 6 to 10 week old male or female Rag2^-/-^ mice.

## Supplementary Material

Supplementary Materials

## Figures and Tables

**Table 1:** Summary of gRNAs used for each approach and the corresponding rAAV6 HDR donor.

gRNA name	gRNA sequence	gRNA target in *CTLA4*	Donor name	Donor summary	Donor use
gRNA 1	GAUGUAGAGUCCCGUGUCCA	Mid exon 2 p. T124P c. 370A>C locus	Donor 1	HA-P2A-GFP-WPRE-HA	Demonstrate editing at exon 2 p.T124P c.370A>C locus
gRNA 2	GATGTAGAGTCCCGGGTCCA	Mid exon 2 p. T124P c. 370A>C locus	Donor 2	HA-codon divergent exon 2-HA	Repair of p.T124P c.370A>C mutation
gRNA 3	UGGCUUGCCUUGGAUUUCAG	Early exon 1	Donor 3	HA-CTLA4-P2A-GFP-WPRE-HA	Universal editing strategy with insertion of replacement sequence in early exon 1.
gRNA 4	AGCUCCGGAACUAUAAUGAG	3’ end of intron 1	Donor 4A	HA-SA-Exons 2, 3, 4 -P2A – GFP-WPRE-HA	Universal editing strategy with insertion of replacement sequence in intron 1 to avoid indels in coding DNA.
			Donor 4B	HA-SA-Exons 2, 3, 4 -P2A – GFP-3’UTR-HA	Universal editing strategy with insertion of replacement sequence in intron 1 to avoid indels in coding DNA.
gRNA 5	GGUCUUGGAAACUAAGCCUG	3’ end of intron 1 in murine *CTLA4*	Donor 5	HA-SA-Exons 2, 3, 4 (murine) - P2A – GFP-WPRE-HA	Intronic editing to restore CTLA4 in murine *CTLA4^-/-^* cells.

## One Sentence Summary

Our results demonstrate the feasibility of a novel therapeutic approach using T cell gene therapy for CTLA4 insufficiency, a devastating inborn error of immunity (IEI) with few treatment options.
