# Supplementary material for: Therapeutic gene editing of T cells corrects CTLA4 insufficiency
Source: Sci Transl Med. Author manuscript; Available in PMC 2025 Jul 5. (PMC7617859; doi:10.1126/scitranslmed.abn5811)
Supplement: Supplementary Materials [file EMS206518-supplement-Supplementary_Materials.pdf]

Supplementary Materials

Supplementary Figure 1: Editing the CTLA4 locus and correction of point mutations

Supplementary Figure 2: Universal editing approach supplementary data

Supplementary Figure 3: Editing the Treg fraction alone

Supplementary Figure 4: Stimulation and cytokine staining of edited cells

Supplementary Figure 5: Lentivirus gene addition approach

Supplementary Figure 6: Assessment of T cell GT for CTLA4 insufficiency using an in vivo murine model

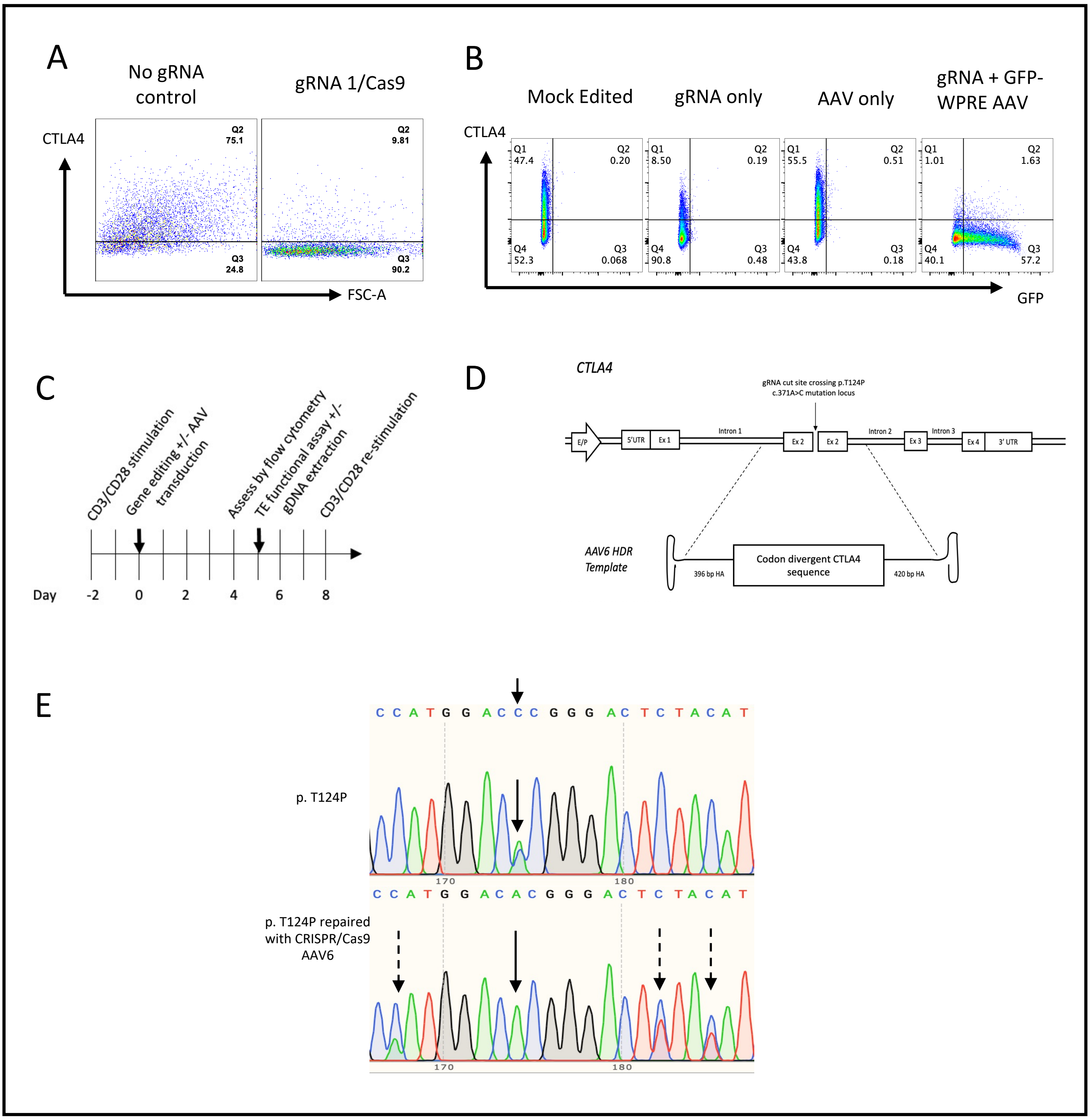

Supplementary Figure 1: Editing the CTLA4 locus and correction of point mutations: *(A) FACS plots demonstrating reduction in CTLA4 expression following nucleofection of gRNA 1/Cas9 RNP to wild type CD4+ T cells. (B) Example FACS plots demonstrating mock nucleofection (Cas9 only)(far left), gRNA +Cas9 (no AAV6) (centre left), AA6 HDR donor only (centre right) and GFP transgene expression in the far-right plot following nucleofection with RNP followed by AAV6 transduction. (C) Experimental timeline for editing experiments. Nucleofection of gRNA/Cas9 ribonucleoprotein complexes was followed immediately (<15 mins) by AAV6 transduction. Phenotypic characterisation of edited populations +/- functional assays and DNA extraction took place >72 hours post nucleofection. (D) Schematic representation of the codon divergent CTLA4 HDR repair template for correction of the p.T124P c.371A>C mutation. (E) Sanger sequence traces from unedited p.T124P c.371A>C cells (top) with the black arrow highlighting the heterozygous mutation. Following editing (bottom trace) the wild type sequence has been restored and the codon divergent repair template has introduced new mutations in the population (black arrows with dashed line).*

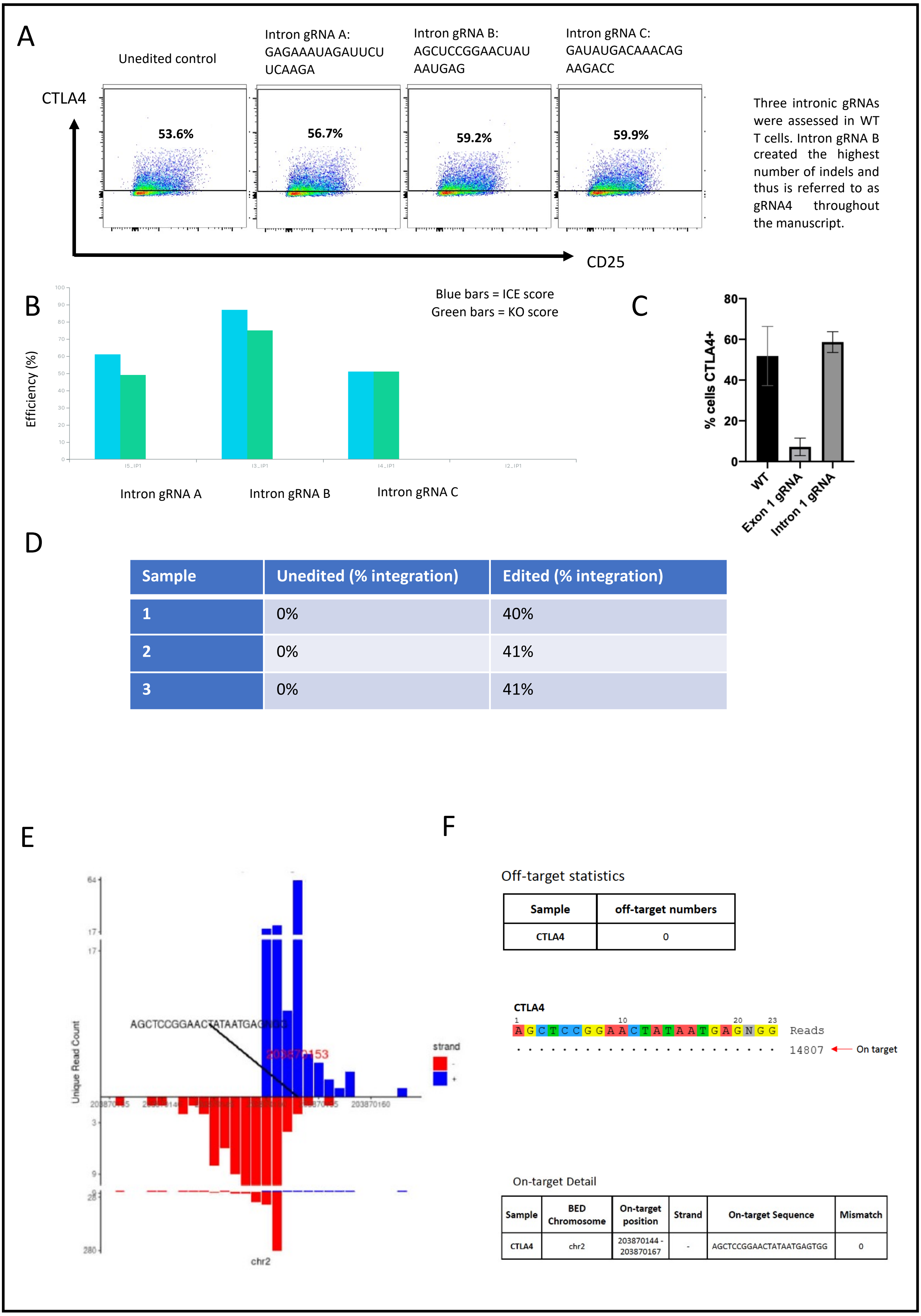

Supplementary Figure 2: Universal editing approach supplementary data: *(A) Representative FACs plots demonstrating normal CTLA4 surface expression following nucleofection of 3 different gRNAs targeting intron 1. (B) Results of the ICE analysis demonstrating the presence of indels in these same population of cells. The blue bars represent the ICE Score - The editing efficiency (percentage of the pool with non-wild type sequence). In the ICE algorithm, potential editing outcomes are proposed and fitted to the observed data using linear regression. The green bars represent the knockout (KO) score - the proportion of cells that have either a frameshift or 21+ bp indel. (C) Summary of experiments (n=3) showing the difference in surface CTLA4 expression between wild type unedited cells, cells edited with gRNA 3 (exon 1) and gRNA 4 (intron 1). (D) % integration calculated from ddPCR results following editing with gRNA 4 and donor 4. (E) GUIDE-seq analysis data showing on-target nuclease specificity of gRNA 4 and (F) off target specificity. (G) Schematic diagram showing the positions of the three different heterozygous mutations used in the experiments to correct T cells from patients with CTLA4 haploinsufficiency.*

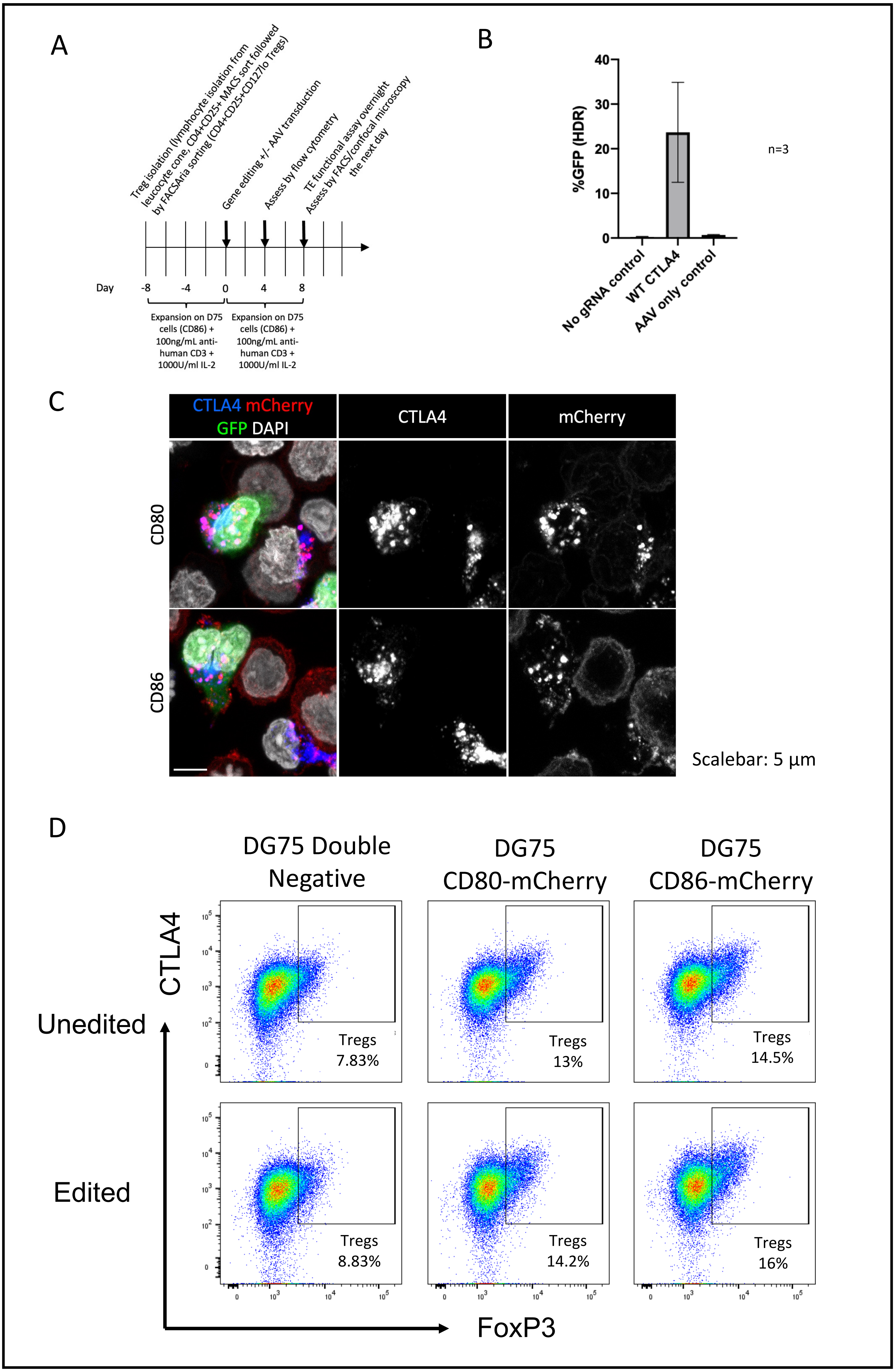

Supplementary Figure 3: Editing the Treg fraction alone: *(A) Schematic of experimental protocol for expansion and editing of Tregs. (B) HDR rates in healthy donor Tregs across three separate experiments from three healthy donors. (C) Confocal microscopy images of edited Treg (green and centre) following 6 hour TE. Co-localisation of CTLA4 with ligands CD80-mCherry and CD86-mCherry is observed.*

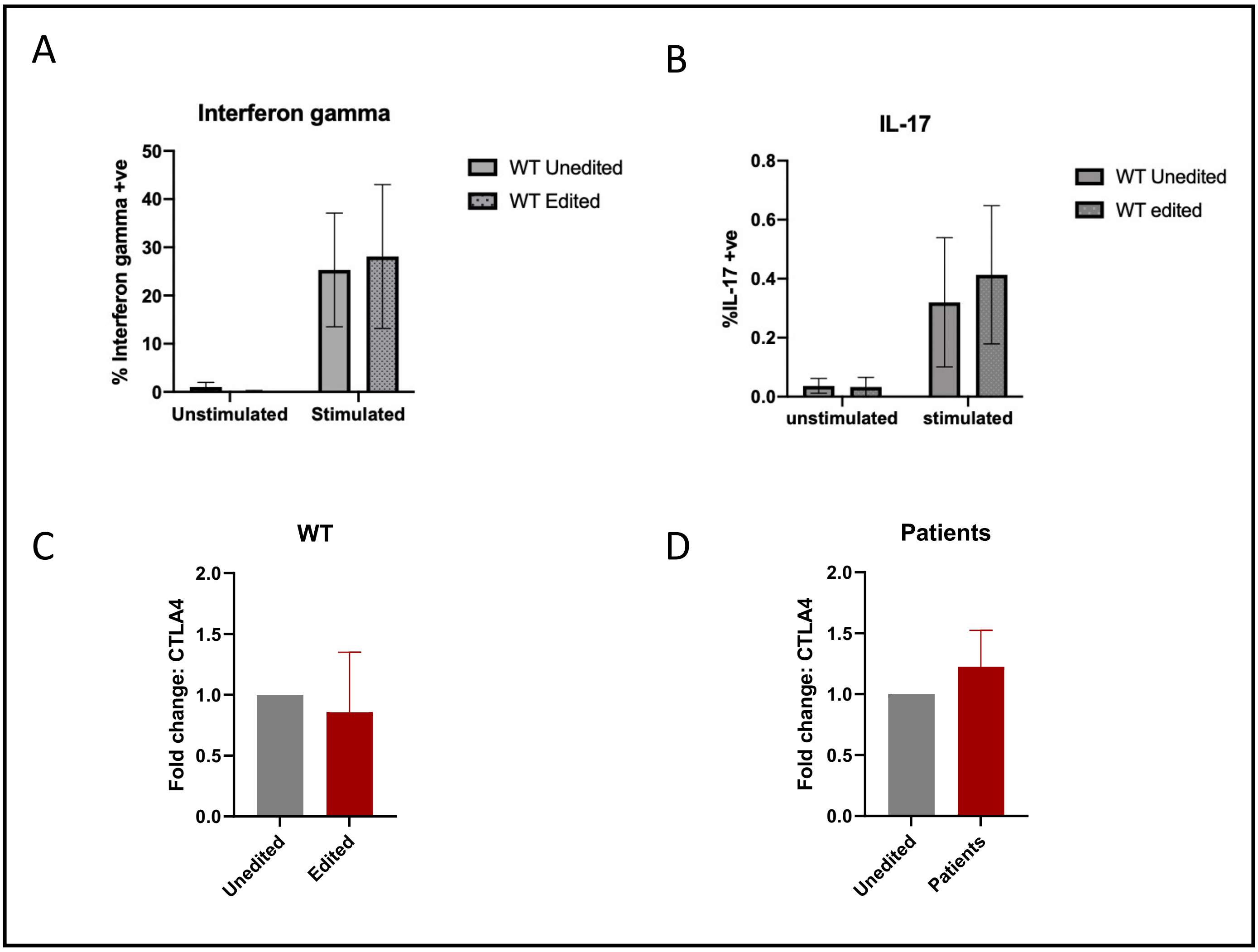

Supplementary Figure 4: Stimulation and cytokine staining of edited cells: *(A) Interferon gamma production (% cells positive) in unedited and edited CD4+ T cells (n=3). (B) IL-17 production (% cells positive) in unedited and edited CD4+ T cells. (C) CTLA4 mRNA was assessed by RT-PCR in WT and (D) patient (left) unedited and edited T cells. Fold changes were calculated using the ΔΔCt method normalized to the levels of GAPDH and relative to unedited control.*

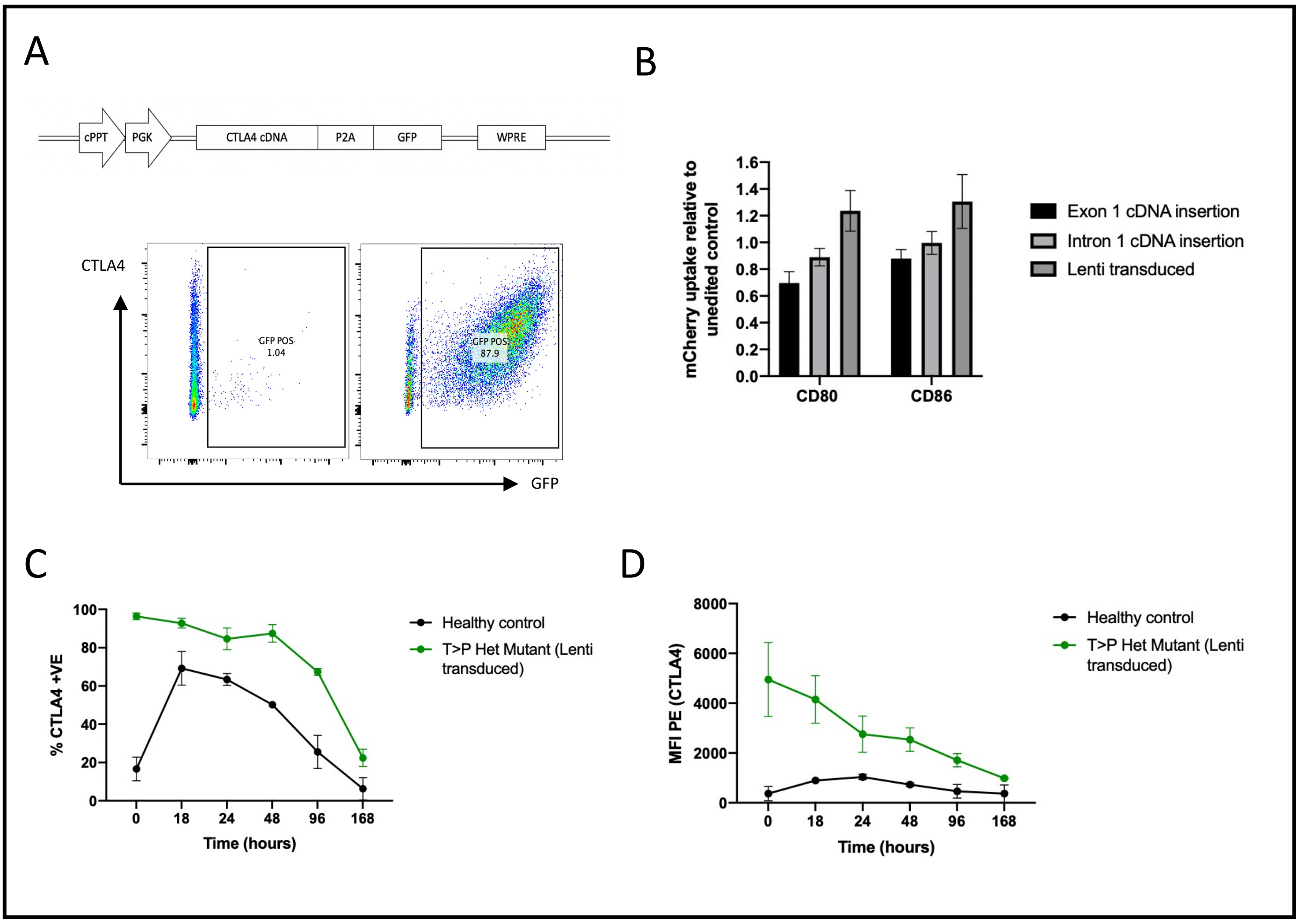

Supplementary Figure 5: Lentivirus gene addition approach: *(A) schematic representation of the PGK-CTLA4-P2A-GFP lentivirus vector (top) and representative FACS plot showing GFP expression following transduction of CD4+ cells with this vector. (B) Results of overnight TE assay with DG 75 cells expressing CD80-mCherry and CD86-mCherry demonstrated increased TE of ligand in the lentivirus transduced cells compared to wild type CD4+ T cells and edited CD4+ T cells. mCherry uptake relative to the unedited control in healthy CD4+ cells (gRNA 3 + donor 3 mean = 0.79, SD = 0.13, n=3, gRNA 4 +donor 4 mean = 0.94, SD = 0.08, n=3, lenti transduced mean=1.27, SD=0.05, n=3). (C) Percentage CTLA4 and (D) MFI of CTLA4 over time following stimulation demonstrating the difference in CTLA4 expression kinetics between lentivirus transduced cells and untransduced healthy control CD4+ cells.*

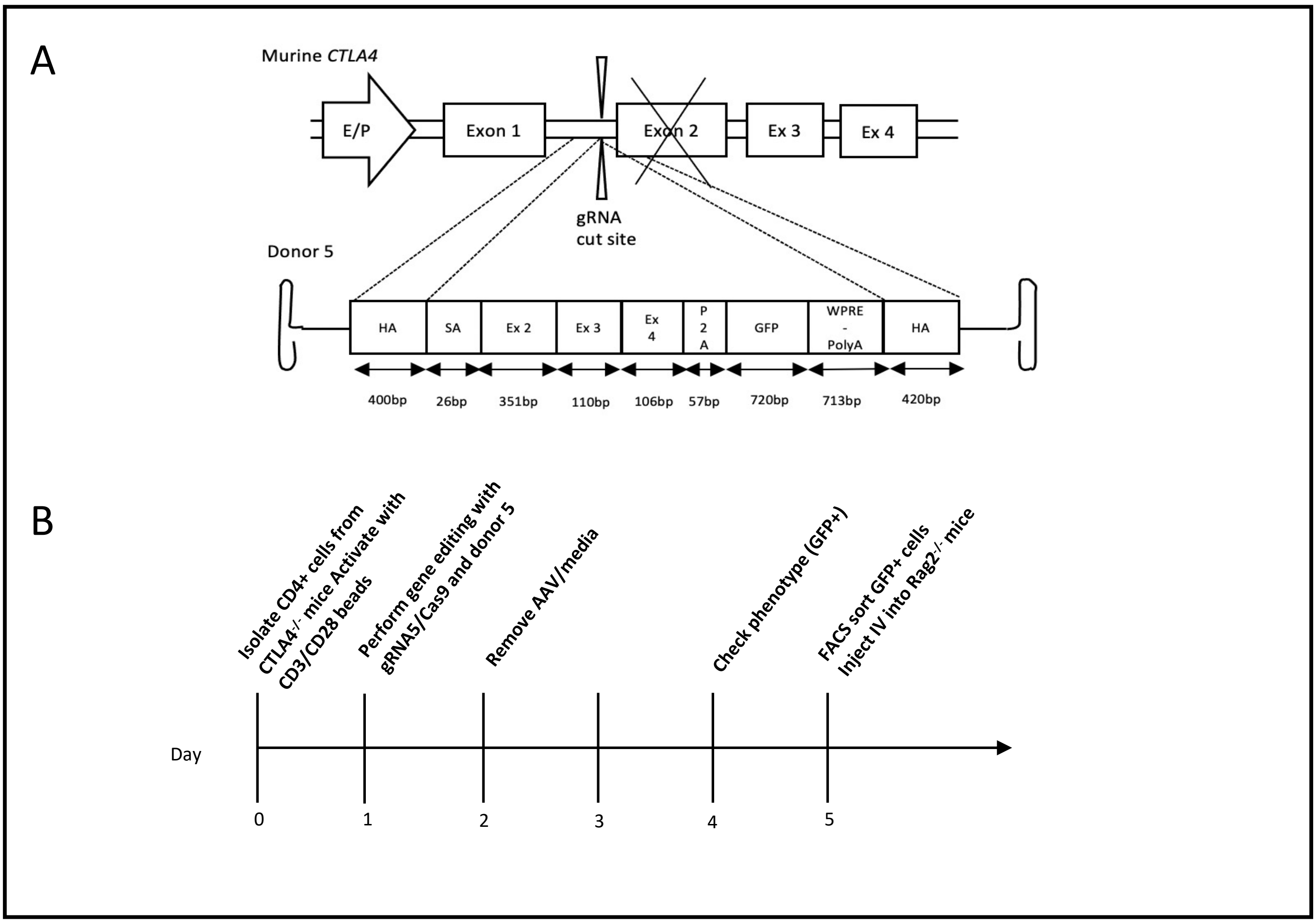

Supplementary Figure 6: Assessment of T cell GT for CTLA4 insufficiency using an in vivo murine model: *(A) Schematic representation of the murine T cell intronic editing strategy (donor 5 HA-splice acceptor-murine CTLA4 exons 2, 3, 4-P2A-GFP-WPRE-HA). (B) Schematic representation of the murine T cell editing protocol.*
